# Model suggests potential for *Porites* coral population recovery after removal of anthropogenic disturbance (Luhuitou, Hainan, South China Sea)

**DOI:** 10.1038/srep33324

**Published:** 2016-09-13

**Authors:** Meixia Zhao, Bernhard Riegl, Kefu Yu, Qi Shi, Qiaomin Zhang, Guohui Liu, Hongqiang Yang, Hongqiang Yan

**Affiliations:** 1 Key Laboratory of Marginal Sea Geology, South China Sea Institute of Oceanology, Chinese Academy of Sciences, 164 West Xingang Road, Guangzhou 510301, China; 2 National Coral Reef Institute, Department of Marine and Environmental Sciences, Nova Southeastern University, 8000 North Ocean Drive, Dania, Florida 33004, USA; 3 Coral Reef Research Center of China, Guangxi University, Nanning 530004, China

## Abstract

Population models are important for resource management and can inform about potential trajectories useful for planning purposes, even with incomplete monitoring data. From size frequency data on Luhuitou fringing reef, Hainan, South China Sea, a matrix population model of massive corals (*Porites lutea*) was developed and trajectories over 100 years under no disturbance and random disturbances were projected. The model reflects a largely open population of *Porites lutea*, with low local recruitment and preponderance of imported recruitment. Under no further disturbance, the population of *Porites lutea* will grow and its size structure will change from predominance of small size classes to large size classes. Therewith, total *Porites* cover will increase. Even under random disturbances every 10 to 20 years, the *Porites* population could remain viable, albeit at lower space cover. The models suggest recovery at Luhuitou following the removal of chronic anthropogenic disturbance. Extending the area of coral reef reserves to protect the open coral community and the path of connectivity is advisable and imperative for the conservation of Hainan’s coral reefs.

Worldwide, coral reefs have been severely impacted by various types of natural and anthropogenic disturbances^1^,^2^,^3^,^4^ and the coral reefs of China are no exception^5^,^6^. Deterioration began decades ago from over-fishing, disease, crown-of-thorns starfish (*Acanthaster planci*) outbreaks, pollution and stress-factors related to climate change, such as hurricanes and bleaching events^3^,^7^,^8^,^9^. For example, the Great Barrier Reef (Australia) has seen average living coral cover decline from ~50% to ~20% from 1960–2003^1^,^9^, and Caribbean coral cover decreased from ~50% to ~10% from 1977–2001^10^. Since the 1998 coral mass mortality and the predicted increase in the frequency, intensity and duration of disturbances, more attention has been focused on declining trajectories and the extinction possibility of coral reefs. But coral reefs are dynamic ecosystems with intrinsic adaptability and resilience and therefore opportunities for mitigation of damage under natural or managed conditions exist. Many instances of recovery from serious disturbances have been observed through recruitment and regrowth^11^,^12^,^13^,^14^,^15^.

To manage coral reefs, trajectory information is frequently desired by conservation authorities, but more often than not, the data environment is poor due to irregular, sporadic, or no monitoring at all. In the present paper we present the derivation of trajectory information, based on inverse solution of transition matrices and simulation of future trends therewith, from a basis of less-than-ideal monitoring data. Since skeletons of scleractinians provide the rocky framework that builds coral reefs, the dynamics of these ecosystems are influenced by changes in the population structure of corals. Traditional monitoring of coral cover alone does not always provide the information needed to predict future trends^16^,^17^, because cover is primarily determined by large colonies and less sensitive to variation in juvenile abundances^18^,^19^, which are key for population dynamics. Consequently, size-distributions of coral populations are a useful tool for assessing status and trends of coral reefs, since they reveal variation in patterns of juvenile input, mortality, and growth among coral species, and among populations of a single species over space and time^20^,^21^,^22^. Population models based on such size-frequency data not only can be used to explain previous population fluctuation but also to predict future population development under hypothetical scenarios^22^,^23^,^24^. Therefore, these models play an important role in resource management^25^.

The South China Sea (SCS) has extensive coral reef development^26^ (at least 8,000 km^2^). Luhuitou reef on Hainan (Fig. S-1) is a typical fringing reef impacted by many human activities. It suffered dramatic decline in coral cover from ~80–90% in the 1960 s to only ~12% in 2009^6^. Reef rock excavation, destructive fishing, mariculture, coastal construction, and local tourism were the main culprits. Establishment of a marine reserve in 1990 reduced frequency and intensity of disturbances and a recent study of the age structure of massive *Porites lutea* showed the majority of colonies to be <50 years old, with 55% of colonies on the reef flat potentially having recruited after establishment of the marine reserve^27^. This suggests two things that can be explored theoretically. Firstly, recruits may have been delivered from a connected reef outside the protected area, and secondly that abundance of smaller and younger colonies may provide the recovery potential for the coral communities following the relatively recent removal of chronic anthropogenic disturbance. Conservation authorities asked for information whether the reef was likely to recover and whether more protected areas were needed. Thus, although no standardized monitoring data existed, it was decided to use and adapt all data in order to build a model that could inform conservation.

In this study we (1) describe the demographics and dynamics of the massive *Porites lutea* during 2010–2014 at Luhuitou fringing reef, (2) build a matrix population model based on the size-frequency data to present the dynamics of massive *Porites lutea*, (3) project the population sizes of massive *Porites lutea* and total live coral cover of this reef over the next 100 years, (4) verify whether and over what period of time such coral populations could return to something comparable to pre-disturbance state, (5) evaluate if the coral populations maintain themselves under various disturbance scenarios.

## Results

### Observed patterns

**Figure 1:**
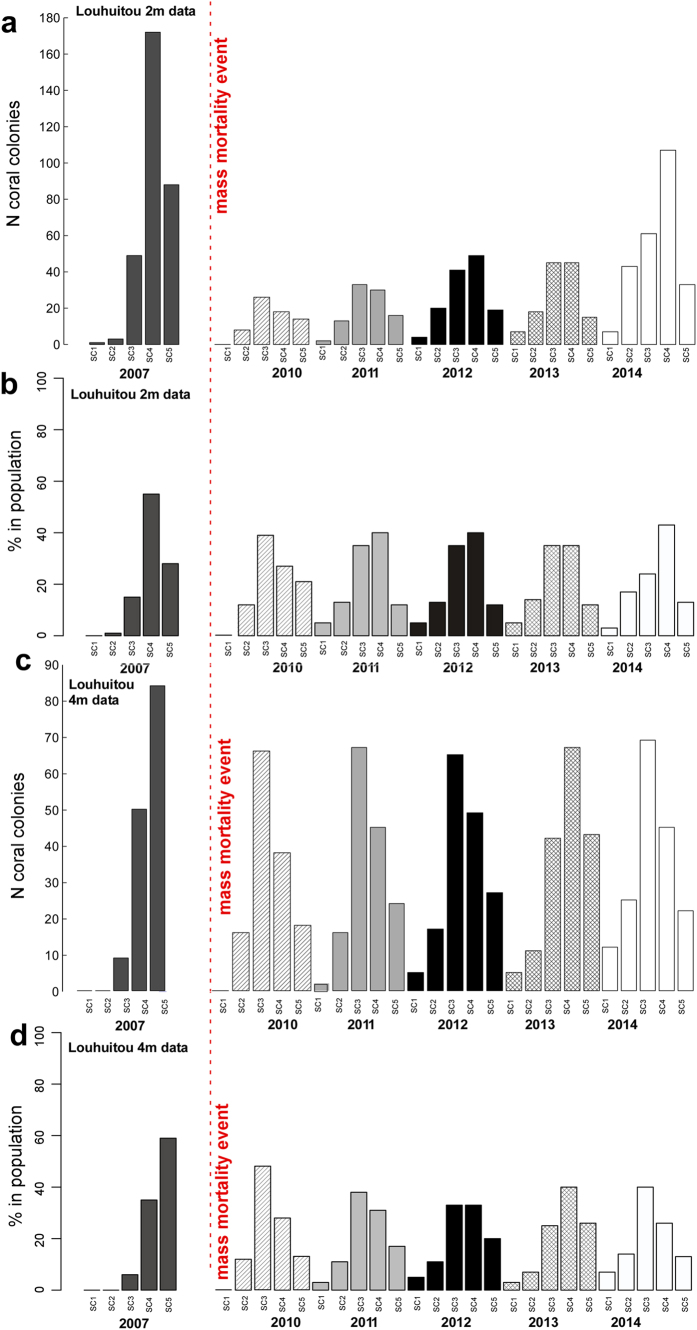
Size-distributions across the surveys (2007, 2010, 2013, 2014). Data from 2012 and 2013 are imputed. (**a**,**c**) Colony counts standardized to area of six phototransects (300 m^2^). (**b**,**c**) Proportional contributions to colony frequency in each survey. These were the data used for the modeling exercise. The severe mortality caused by the 2010 event, and subsequent regeneration with changed population dynamics, are clearly visible.

According to the standardized data of colony counts per size class (SC1: 0–5 cm, SC2: 5–10 cm, SC3: 10–20 cm, SC4: 20–40 cm, SC5: >40 cm diameter) and its proportional contributions to colony frequency at two depths of Luhuitou reef (Fig. 1), there was a dramatic decline from 2007–2010 in coral abundance at 2 m depth (313 vs 66), but less so at 4 m depth (143 vs. 138), where there was a sharp decrease in the proportion of the largest size class (SC5: 59% vs 13%). This was most likely attributable to a severe mortality event associated with nearby major harbor construction (2010–2011) and potentially also exacerbated by the hot 2010 summer (Fig. S-2). Dredging operations during early phases of construction of the Midhill Peninsula Sailing Club caused severely elevated turbidity and sedimentation. Additionally, the average monthly temperatures in summer 2010 exceeded those of all previous years except 1998 (Fig. S-2). The combination of acute sedimentation stress from nearby port dredging, construction on the reef itself, and abnormally high summer temperatures set the environmental conditions required for a major coral die-back.

Although mortality was not followed over the stress period, it is evident that the decline in numbers and the changes in size-distributions of corals should be correlated with this event and that the regenerative period should be correlated with the cooler years that followed after dredging had ceased as well. In the years following the mortality of 2010, regeneration seems to have occurred across all size-classes at 2 m, demonstrated by total coral abundance and proportion of larger size-classes generally following an increasing trend (Fig. 1). At 4 m, a similar trend existed until 2013, but in 2014 small and medium corals (SC1-3) again increased, potentially suggesting renewed recruitment and strong survival of small size classes into the larger classes.

The proportional size distribution of corals prior to and after the mortality event differed, with the largest two size-classes clearly dominating the pre-disturbance coral population (Fig. 1). During the regeneration phase, smaller size classes first predominated and only in the fifth year after disturbance at 2 m and the fourth year at 4 m did larger size-classes (SC4 + SC5) again become increasingly prevalent. This is congruent with the coral’s growth rate (11.3 ± 0.2 mm^−1^ yr^−1^)^27^. Interestingly SC1, which likely includes recruits from about 1–4 years of age as well as remnants of larger corals that had lost tissue, failed to grow or had undergone fission^13^, did not increase dramatically. We therefore have no indication of a recruitment pulse as the basis of the regenerative phase. SC3 had the highest proportion in the population in 2010 and declined in relative frequency in the following years (but not in absolute numbers, Fig. 1). This pattern suggests that regenerative growth, replenishing the larger size-classes, may have originated from SC3. Expansion of the larger size-classes was likely achieved at least as much from regenerating colonies as by new recruits. Purely recruitment-driven regeneration would require a larger contribution of SC1 than was observed. In post-2010, there were more small colonies than in 2007 and 2010, suggesting recruitment into recently vacated space. A higher pre-disturbance proportion of corals in SC5 at 4 m suggested a somewhat less disturbed environment with larger and older corals.

The 2010 event served to reduce the relative abundance of the largest two size classes (>20 cm diameter). While our data cannot conclusively address the mechanism, this could have occurred by wholesale mortality, mainly of large colonies, or by partial tissue mortality. Shrinkage into smaller size-classes is frequently the case during bleaching events, and can lead to noticeable increases in the smaller size-classes^13^,^22^. A strong recruitment pulse would have bolstered SC1 and, only in subsequent years SC2 and SC3.

**Figure 2:**
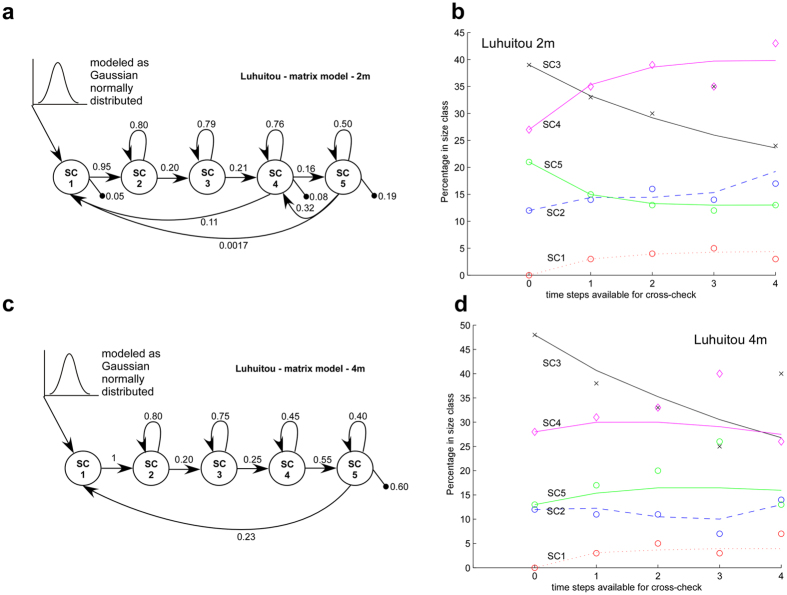
Life-cycle graph of the applicable growth model corresponding to the matrix model inversely solved by Wood’s method (**a**,**c**). Several models were tested, those illustrated were most successful in hindcasting data. SC = size class, arrows denote growth transitions from smaller to larger size class, or recruitment from larger into smallest size-class. Arrow from the outside represents recruitment from a connected population. Line with dot signifies death. Note that in a Leslie matrix, fertilities and growth-transitions need not add to unity. (**b**,**d**) successful hindcast and model verification. Circles, diamond and x’s are observed datapoints, broken and solid lines are model-based hindcast. To optimize model fit, a recruitment sequence of [5,0,0,3,0] was added. Each value represents the number of recruits that were added at the respective time-step (i.e. 5 at step 1, 0 at step 2, etc.).

2 m-data showed major differences from 4 m-data, especially pre-disturbance, which might have been based on different life-dynamics of the corals due to their position in distinct geomorphological zones of the reef (slope versus crest). Therefore, the two data sets were evaluated separately. From both size distributions, two distinct matrices of growth transitions could be extracted (Fig. 2).

### Growth models

Several potential matrix models were inversely solved from standardized proportional abundances, with a variety of shrinkage, loop and death options, then hindcast against known datapoints and evaluated for fit. The final models as life-cycle graphs and verification by hindcasts are shown in Fig. 2. Strong growth transitions exist in the small SCs, increasing retention (loops) in the large SCs, and little local recruitment (Fig. 2a,c). Therefore, since recruitment from the outside could be expected as well (many coral populations are demographically open), it was necessary to add a vector of recruits (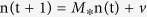), where υ has a value in position 1 and else consists of zeros, the empirically-obtained values at position 1 were υ_1_(t = 0…4) = [5,0,0,3,0]; each value represents the number of recruits that were added at the respective time-step (i.e. 5 at time t = 0, 0 at time t = 1, etc.)) to obtain an optimal fit in the hindcast (Fig. 2b,d). The hindcast trajectory started with vector n(t = 0) (count of 2010, which had not been used in inverse solution of the transition matrix) and solution vectors n(t = 1…4) approximated the size distributions obtained in the field (2011–2015), from which the matrix *M* had been solved, very well (2 m: R^2^ = 0.938, 4 m: R^2^ = 0.895). This suggests that the inversely solved transition matrix was indeed realistic, with the refinement that recruitment into the population should be a mixture of local and primarily imported.

Obtained with the same process as the growth-model for the 2 m data, the fit of the 4 m model was less precise, due to a variable trend in SC3 and 4. The model hindcast the overall trajectory well under the same recruitment assumptions, requiring addition of vector υ(t = 0…4) (see above) to improve the fit. This suggested that at 2 and 4 m the recruitment process might be similar and, most of all, require larvae import from connected populations. This seems plausible since the two sampling strata were close enough to be within a uniform hydrodynamic regime.

The density-dependent forecast model gave the most credible results with a parameter b = 0.001 in SC3, 4, and 5; and addition of Gaussian normal recruitment of mean and variance 25 (by adding a vector with a Gaussian random number in position 1) to SC1. This approach allowed to leave the solved transition matrices (Fig. 2) untouched. Thus, these models assume very low local recruitment (Fig. 2) and a preponderance of imported recruitment, suggesting a largely open population. This assumption is concordant with observations in the literature and other modelling approaches that stress the importance of open larval movement (among reefs) in coral populations.

**Figure 3:**
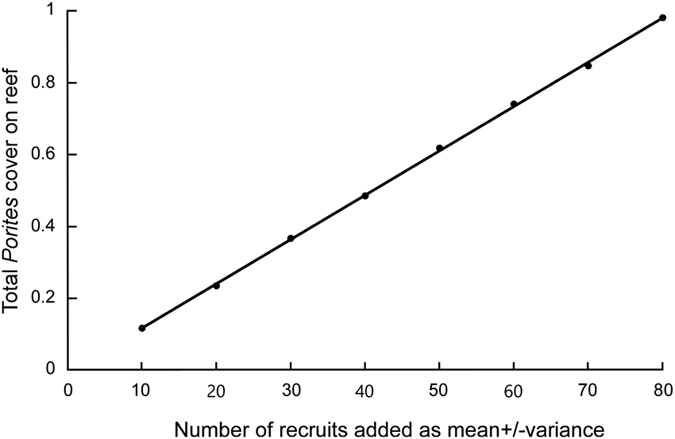
The relationship between number of recruits and total *Porites* cover reached in models at Luhuitou reef.

**Figure 4:**
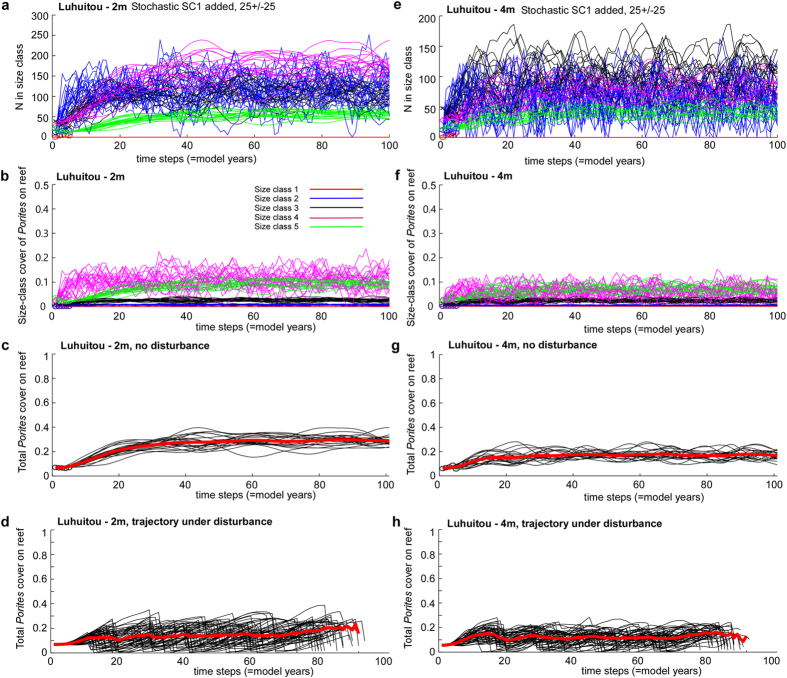
The density-dependent growth model of corals at 2  and 4 m depth on Luhuitou reef. (**a**,**e**) Frequency of size classes, if a Gaussian random recruitment of 25 +/− 25 is added at each step. (**b**,**f**) Coral cover in each size class as share of the total are of six transects. To obtain total cover on the reef, the proportions of all size-classes must be summed (**c**,**g**). The effect on *Porites* space-cover of disturbances killing randomly between 0–100% of the population in random intervals between 10–20 years (**d**,**h**). Shown are 50 of 500 realizations only, for means of graphical clarity. The thick red line is the mean of all trajectories. It shows that despite strong variability, good space cover of *Porites* can be maintained.

The results clearly indicated that the model was highly sensitive to recruitment assumptions (Fig. 3). There was a linear relationship between recruitment level and total calculated cover of *Porites*. The more recruits imported from other connected populations, the higher total cover could be achieved. *In situ* measured recruitment by all species into the whole coral community ranged from 0.29 to 1.08 recruits. m^−2^ (The Bulletin of Marine Environment Quality of Hainan, 2004–2009). This was about equivalent to the most likely scenario of regeneration employing Gaussian random recruitment (mean and variance 25 per transect) at each step (Fig. 4), which would range from 0 (all negative values were set to zero, since no negative recruitment is possible)-0.4 *Porites* recruits.m^−2^. Since *Porites* made up between half to a third of cover in the undisturbed community, this upper value (0.4) was considered a realistic compromise.

Under this scenario, it would take about 40 years from the disturbance until distribution of size classes and cover would settle down within a stable regime at both depths (2 and 4 m; Fig. 4). Total *Porites* cover in the outcome of this scenario ranged in about 60 years from 20–30%, with only slightly higher cover at 2 m. As should be expected, around the equilibrium, the largest size classes would dominate cover. SC4 is expected to be the most frequent coral size-class at 2 m, overtaking SC2 after 30 years (Fig. 4a). Thus, after about 40 years, the reef at 2 m should begin to be dominated by large coral colonies of SC4 and SC5 (Fig. 4b). After about 60 years, the overall *Porites* cover should have settled around a stable value (Fig. 4c). At 4 m, it is expected that while coral cover will be almost the same as at 2 m, the population will be characterized by smaller corals, since SC3 will remain numerically dominant (Fig. 4d). The largest SC5 corals will be the most important contributors to the total *Porites* cover (Fig. 4e), which should be lower at 4 m than that at 2 m (Fig. 4g).

It must be noted that these dynamics will only be valid if no further disturbances occur over the next 100 years, which is unlikely. Population trajectories were also predicted under disturbances at random intervals between 10 and 20 years (a likely time-frame, given the recurrence of hot years (Fig. S.2; 1987/88, 1998, 2010) and also disturbances in other high-latitude systems^13^), using the same population parameters as in the regeneration scenario. The results suggested that this *Porites* population could maintain a viable population, albeit at lower space cover, due to the open population structure with a strong connectivity to outside larvae sources (Fig. 4d,h). Both the projected population dynamics under absence of further disturbances and random disturbances suggested that the open *Porites* coral populations of Luhuitou reef can indeed increase and potentially return to pre-disturbance levels.

## Discussion

### Model of massive *Porites* suggests potential regeneration of coral community at Luhuitou fringing reef

Luhuitou reef suffered dramatic decline in coral cover from ~80–90% to 12% from 1960 s–2009. Coral community structure also changed dramatically into the current predominance of massive *Porites*^6^ (primarily *P*. *lutea*). Recent studies of age distribution in *Porites lutea* at this reef found an abundance of younger colonies indicating a potential upward trajectory, a hopeful sign of potential recovery, following the establishment of Sanya National Coral Reef Natural Reserve in 1990^27^. Our models of massive *Porites* suggest that, given sufficient recruitment, this population can maintain an upward trajectory.

Population dynamics of stony corals may reveal processes of change in the community structure of coral reefs^17^, so massive *Porites*, as a major and dominant coral at Luhuitou, could serve as a suitable indicator of reef condition. Size-frequency distributions are often used for describing population processes and environmental effects^10^,^19^,^28^, since they can reveal variation in patterns of juvenile input, partial mortality and longevity. Our model suggested that the increase of small *Porites* was mainly achieved by new recruitment (as opposed to shrinkage^22^), and, as long as that was maintained, corals would recover. Potential population growth was also observed in other corals. For example, many small branching *Montipora* colonies began to appear at some reef flat sites during our field monitoring surveys.

It may seem contradictory that our model suggests an increasing trajectory of coral populations while the live coral cover in the region remains at overall low level. Mean coral cover is commonly used in the assessment of coral reef health^29^. But percent cover of corals is often determined primarily by large colonies and is less affected by changes in the densities of small size classes^17^. Even in studies where small submassive coral colonies were particularly abundant, they did not make a large contribution to total cover^19^. It is this very component that may be missing in many studies.

### Limitations and caveats

While realistic, the presented model is built on a basis of data that leave much to desire. But it was this dearth of replicated and reliable data, faced with a distinct need of trajectory information to aid nature conservation authorities that prompted model development. Since model development was not planned *a priori*, no suitable sampling regime had existed. Data were obtained by different investigators at different sites and at different effort levels. In 2010, half as many samples (N = 3) existed as in 2013/14. Since corals are sessile, constant detectability was assumed, corrections^30^ were foregone, and counts from that year were doubled. This approach may prove problematic once variation in detectability due to, for example density variability, becomes known. To fill gaps in data (2011, 2012) needed for annual frequency estimates, regression imputation^31^ was employed. The linear model used here may eventually prove inadequate in longer monitoring time-series. Above challenges, combined with the fact that samples were not taken in the same areas, did not allow use of actual counts to solve the transition matrix from the available monitoring size-class distributions. To deal with this problem we resorted to express coral frequency as “standardized proportional frequencies”^22^ (see Material and Methods). This approach assumes that although the corals measured among years were not the same, they nonetheless formed part of a uniform population that in its entirety was assumed to follows the same transition rules. While this assumption cannot be proven with our data, it is a tacit assumption implicit in most matrix models throughout the literature^32^. If trajectory information is preferred over predicted number of colonies, then counts in the population vector can be transformed into proportional contributions by each size class (see Material and Methods). While this changes the size of the vector, it does not change its direction and will therefore deliver equivalent trajectory (i.e. directional) information. In the case of our model, this approach is justified, since we were interested not in exactly how many corals would occur in what area, but whether coral cover in general, in an area encompassed by six phototransects (300 m^2^) would increase or decrease and under what scenario. To this end, we later transferred coral size information into coral cover, by summing the areas contributed by each size class. These generalizations allowed to inversely solve transition matrices, but these solutions suggested very low local recruitment (i.e. transitions from the larger SCs into the smallest SC). The best hindcasts could be obtained if recruits were added during each model year, which is a plausible approach since many coral populations are open^33^. Based on this observation, a random number of recruits was added each year during following trajectory simulations, the amount tuned to known recruitment level. While certainly plausible, this made the model highly sensitive to these recruitment assumptions, making classical eigenanalysis^32^ of the solved Leslie matrices obsolete and moving the exercise firmly into the realm of matrix-based simulation.

### The trajectory of Luhuitou fringing reef over next 100 years

Population dynamics of scleractinian corals may reveal processes of change in the community structure of coral reefs^16^,^22^, so massive *Porites*, as a dominant coral at Luhuitou, could be a suitable indicator of reef condition. Other coral population or community models from the Red Sea^34^,^35^, Indo-Pacific^36^,^37^,^38^, Carribean^17^,^18^ and Great Barrier Reef^39^,^40^ mainly focus on the effect of non-anthropogenic disturbances (*Acanthaster planci* predation, bleaching, storms), but Luhuitou reef declined dramatically over the past 50 years mainly due to local anthropogenic impacts^6^. Since Luhuitou reef is adjacent to Sanya City and Harbour, it plays an important role in providing coastal defense, seafood, recreation, and many other goods and services for local residents and tourists of a urban population that increased from <70,000 in 1984, to >260,000 in 2007 (Sanya Statistic Department, 2008). From the 1970 s–80 s, coral block mining, overfishing, and destructive fishing (blast, cyanide, and electric fishing) resulted in severe coral mortality and damage to the reef’s structure^6^,^36^,^37^. Since the Sanya National Coral Reefs Nature Reserve was established in 1990, such destructive activities have been reduced significantly. However, increased mariculture (pearl oyster, *Eucheuma*, prawn, and abalone) in the 1990 s and diving activities (increasing since 2004) still impart stresses and killed most *Acropora* and *Pocillopora*^6^. Massive *Porites* persisted due to their high resistance and adaptability to disturbances^27^.

Many massive *Porites* recruits survived and grew up under these conditions. Age structure in 2007 suggested that about 55% of *P*. *lutea* on the shallow reef flat had recruited after establishment of the reserve, compared with 18.4% of *P*. *lutea* at 2 m depth and 6.1% at 4 m depth on the reef slope^27^.

Our model shows that size frequency will change towards predominance of large size classes and total live coral cover will increase under normal environmental condition. In ~30 years large SC corals will become more frequent than small SCs and *Porites* cover will stabilize between 20% to 30% at 4 m and 2 m depth. This projection was modeled under “normal” conditions, or no further disturbances over the next 100 years. Unfortunately, this desired normal condition may never exist. Although we could take more local measures to protect coral reefs from anthropogenic influences, global climate change will increase frequency, intensity and duration of disturbances^41^. High intensity storms can reduce standing coral colonies to <5% and a steady decline in abundance could be a consequence of increasing storm frequency^42^. Population dynamics of massive corals in the Gulf of Oman under 10-year and 16-year disturbance scenarios indicated return to pre-disturbance coral cover but overall population size could not recover^23^. Also our projections of population trajectories under disturbances recurring randomly in intervals between 10–20 years suggest that Luhuitou *Porites* populations could stay viable, but with lower cover.

### The importance of connectivity for open populations in coral reef protection

Our transition matrices reflect very low local recruitment and preponderance of imported recruitment, suggesting a largely open population of massive *Porites lutea* at Luhuitou. While the coral’s reproductive strategy (broadcast-spawning) makes this not unlikely, more localized stock-recruitment relationships have been observed in other studies^3^,^4^,^15^. Massive *Porites lutea* have a long-lived life history strategy, relatively low rate of sexual recruitment and slower growth rate^40^ compared with other shorter-lived corals, for example, *Acropora* and *Pocillopora*^34^,^41^. In a coral community without disturbances, *Porites* will suffer from competition by fast-growing species until large colonies reach their size-refuge^42^. *Porites* is also relatively resilient toward natural and anthropogenic disturbances, so when the reef was impacted, many *Porites* survived and recruitment from nearby connected populations helped the population to regain momentum and growth, with the hope for eventual recovery.

Connectivity is essential for population survival and regeneration^43^,^44^. Bramanti *et al*.^45^ projected dynamics of *Pocillopora damicornis* under global climate change (GCC) and ocean acidification (OA) assuming closed and open populations. Closed populations were extirpated within 100 yr, while open population, with larval supply of 10% from connected populations could persist regardless of high temperature and OA. Thus, connected populations are important and can act as “coral refuges” or “re-seeding batteries”^13^,^22^,^23^. Once larvae source populations are destroyed or any other factors interfere with connectivity, reef resilience overall would be compromised^23^.

Marine reserves are envisaged as conservation tools and natural laboratories, and therefore many coral reef reserves were established for sustainable management in the past decades. But a majority of existing reserves are small and only encompass part of the metapopulations of their constituent species. Luhuitou reef is no exception. In this respect, it is important to establish larger reserves in the future protection strategy. Extending the area of coral reef reserve could protect more of the open coral community and the path of connectivity, and therefore enhance coral recruitment and adaptation of coral communities to disturbances. We hope that this study can provide rationale for extending protection of Hainan coral reefs beyond the present conservation areas.

## Material and Methods

Luhuitou fringing reef is situated on Hainan Island, northern South China Sea (Fig. S-1). It is protected since the 1990 s and the coral population has been monitored^6^. The present study was prompted by the desire to forecast whether regeneration was possible after the decline in corals demonstrated by Zhao *et al*.^6^ As basis of the analysis, several random 50 × 1 m video-transects were available (2007: 20 transects = 941(2 m) + 428(4 m) corals; 2010: 3 = 33 + 69, 2013: 6 = 130 + 168; 2014: 6 = 251 + 173). Transects were positioned in two distinct geomorphological zone of the reef – at 2-m depth, samples were haphazardly placed on the 0.3 km wide reef crest, while at 4-m depth, samples were situated parallel to the depth-contour on the reef slope. Although situated within a close depth-range, the distance between sampling sites was several hundred meters. The two distinct habitats used to have different coral communities and experienced differently strong impacts in the past^27^,^37^. These transects were used to evaluate the size-structure of the contained corals, arranged into five size-classes (SC1: 0–5 cm, SC2: 5–10 cm, SC3: 10–20 cm; SC4: 20–40 cm; SC5: >40 cm diameter). The four sampling years were evaluated. Coral declined between 2007 and 2010 (during a mass-mortality in 2010), but 2010 (after the mortality) until 2014 was a period of increase. The question was whether any indication could be gained from the interval 2010–2014 that allowed evaluation and prediction of future trajectory. Such a model could then allow a forecast with regards to potential population recovery and the speed towards such a target.

Prior to model development, several issues in data quality had to be overcome. Firstly, in 2010, only half as many transects were evaluated as in 2013, 2014. This was addressed by doubling the colony count in 2010 from three transects to make it more compatible with the counts from six transects henceforth. Furthermore, video transect samples were not repeats among the sampling years, but were taken at random locations. Riegl and Purkis^22^ used transects from random locations around a central georeferenced point to successfully hindcast population matrices, by using “standardized proportional contributions” of corals (i.e. proportion *100; the multiplier could be any number that produces counts > 1), rather than absolute frequency. The same approach was taken here since it is useful to account for differences in the density and number of individuals included in each year’s sampling. It can be justified as follows:

In a matrix model of the form


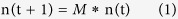


the growth of values in the solution vector n(t + 1) is primarily determined by the values in matrix *M*, with values in vector n(t) variable. If n(t) is changed from actual frequency counts into a vector of proportional contributions of its components, then only its magnitude, but not its direction is changed. Thus, if there is reason to believe that relative size distribution in a sampled population should be uniform across samples regardless of whether more or fewer individuals were present within each sample, then no information is lost by transforming the absolute values in a vector into its proportion (an operation routinely employed when expressing stable distributions as proportional contributions in the eigenvector^32^). This approach allows comparing the size-frequency distribution and dynamics of samples with different sizes, by standardizing their population size (for example, to proportion*100). It further allows use of samples that would normally be highly undesirable, because collected in areas of different densities and/or living cover or, as in the present case, from variable sampling effort.

Another issue was that sampling years were irregular and gaps existed (samples existed from 2007, 2010, 2013, 2014). To increase the amount of data points available and to obtain a data series of yearly samples, we imputed two data points (for the years 2011, 2012). We used regression imputation^31^ that estimated the unsampled attributes from those known by linear regression.

These manipulations of the data provided a series of five annual (2010–2014) vectors of standardized size (proportional contribution of each size-class *100). Using four of these data vectors (n(t = 1) to n(t = 4), excluding n(t = 0)), Wood’s method^32^,^46^ was used to inversely solve the transition matrix (Leslie matrix) responsible for the changes among the annual population vectors within the observed period (2010–2014). The solved Leslie-matrix model is essentially an exponential growth model, and the linear approximation over a short time-period is realistic. The realism of the solved matrix-model was tested by hindcasting model runs against the known and imputed data-points. Hindcasts were started by multiplying the solved size-transition matrix by the first vector of standardized frequency n(t = 0) (year 2010, which was not used for solution of the matrix) and run for four steps. Each solution from these four steps (i.e. the resulting vector, which is a size-distribution), was compared to the actually observed size-distributions from 2011–2014 (Fig. 2). Since model hindcasts started at vector n(t = 0) (not used for solution of the matrices) and from there developed trajectories that needed to approach those actually observed (which had been used for solution of the model but not in the hindcast) this is no tautology.

Several potential matrix models (i.e. with different pathways for growth, shrinkage, fertility) were developed via different constraint matrices^32^ and solved, which were then tested in hindcast for accuracy^32^. The ones fitting best to the observed population trajectory at 2 m and at 4 m, were chosen for further evaluation (Fig. 2).

Steps in the data analysis were:

1. Evaluate video-transects for size-classes.
2. For each sample year, calculate proportion of each size class to total coral frequency.
3. Multiply proportion by 100.
4. Impute data for 2011 and 2012 by regression on all existing datapoints.
5. Develop matrix model structure for life-cycle graph.
6. Solve matrix from transect data using Wood’s method.
7. Hindcast solution and compare to observed data.
8. Add corals to SC1 to correct for low count.
9. Solve until good fit with observed data is achieved.

Step 5 involved the testing of several potentially possible transition matrix structures, which were encoded in the constraint matrix^32^. Only one such transition matrix (Fig. 2) achieved a solution that produced outcomes comparable to observations in the field. The structure of the evaluated model is shown as life-cycle graph in Fig. 2 The model allows for persistence within size classes as loops, growth by one size-class only, shrinkage of SC5 to SC4, and recruitment from SC4 and SC5.

Step 8 was necessary because in photo transects, about 50% of all corals in SC1 are likely to be overlooked^34^, and in video transects, due to the blurry nature of screen grabs, this percentage is likely to be even higher.

The solved transition matrices included the life-stage transitions and recruitment (i.e. transitions from smaller SCs into the larger SC; fertility cannot be measured since gametes and larvae were not counted). A Leslie-matrix type model is characterized by exponential increase, which in our case was not considered realistic since corals are limited by an upper carrying capacity. Furthermore, the Leslie-matrix was solved only for four years, and it could not be expected that long-term recruitment could be adequately estimated. Also, recruitment is notoriously variable in space and time^12^,^43^,^45^,^47^. Therefore, to forecast population trajectories it was necessary to split the Leslie matrix (previously solved by Wood’s method):


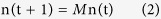


(Where n(t) is the vector of size-frequency observed at time t, and M is the inversely-solved Leslie matrix) into


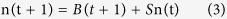


where S is the survival matrix, consisting of inter-stage survival probabilities in the subdiagonal, loops in the diagonal, shrinkage in the upper subdiagonal and else all zeroes. And the matrix of increases per time-step


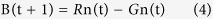


where R is a fecundity matrix with positive entries in the first row (at positions 4 and 5, since all other stages were not considered fecund) and else zeros, G is a matrix of growth-inhibition with entries in the first row only and zeros elsewhere. G was chosen to be a Ricker-function where:


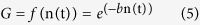


since the exact values of *R* are unknown and in field studies usually unobtainable^32^,^34^, forecasts were performed by using Gaussian random numbers of defined mean and standard deviation (see Results). Also the exact value of the competition parameter b was unknown. It was empirically chosen to allow a population trajectory that would settle at, or near, population characteristics as observed prior to the disturbance events^27^.

If density effects were also allowed to act on the other size-classes, the entire growth model became by combining equations 3, 4, and 5:


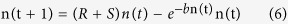


(R + S) represent the Leslie matrix solved by Wood’s method. It was therefore theoretically possible to perform eigenanalysis of the matrix and evaluate the spread of the dominant eigenvalue, as asymptotic growth rate over the stochastic recruitment assumptions. This was, however, not performed in the analyses, since local recruitment, a factor heavily influencing ergodic dynamics, was unknown. Furthermore, we wished to model the population as open, or at least allow for variable degrees of openness^33^. In an open population, the dominant eigen value loses its importance as unique determinant of the intrinsic rate of increase.

Next steps in the analysis were:

1. Insert stochastic recruitment from outside and within the population
2. Insert carrying capacity (vary between size-specific or general) via –*b* (eq.5)
3. Run for 100 years and evaluate when carrying capacity is reached

To optimize model fit, an empirically-derived recruitment sequence of [5,0,0,3,0] was added during the first five hindcast steps. Each value represents the number of recruits that were added at the respective time-step (i.e. 5 at step 1, 0 at step 2, etc.).

To evaluate the population trajectories in a future with disturbances, we used the same population parameters as used for solution of the most likely regeneration scenario and assumed disturbances recurring in random intervals between 10 and 20 years, and killing randomly between 0 and 100 percent of the local corals. We used a uniform random distribution. Any number of repeat model runs are possible (we ran up to 10.000), but graphs show only 50 for reasons of clarity, and the mean of all trajectories was used as indication whether corals would persist or not.

## Additional Information

**How to cite this article**: Zhao, M. *et al*. Model suggests potential for *Porites* coral population recovery after removal of anthropogenic disturbance (Luhuitou, Hainan, South China Sea). *Sci. Rep*. **6**, 33324; doi: 10.1038/srep33324 (2016).

## Supplementary Material

Supplementary Information
