# Supplementary material for: Model suggests potential for Porites coral population recovery after removal of anthropogenic disturbance (Luhuitou, Hainan, South China Sea)
Source: Sci Rep. 2016 Sep 13;6:33324. doi: 10.1038/srep33324 (PMC5020734; doi:10.1038/srep33324)
Supplement: Supplementary Information [file srep33324-s1.pdf]

Supplemental Information

Model suggests potential for *Porites* coral population recovery after removal of anthropogenic disturbance (Luhuitou, Hainan, South China Sea)

Meixia Zhao[1, 2], Bernhard Riegl[2], Kefu Yu[*1, 3], Qi Shi[1], Qiaomin Zhang[1], Guohui Liu[1], Hongqiang Yang[1], Hongqiang Yan[1]

[1] Key Laboratory of Marginal Sea Geology, South China Sea Institute of Oceanology, Chinese Academy of Sciences, 164 West Xingang Road, Guangzhou 510301, China zhaomeix@scsio.ac.cn

[2] National Coral Reef Institute, Department of Marine and Environmental Sciences, Nova Southeastern University, 8000 North Ocean Drive, Dania, Florida 33004, USA rieglb@nova.edu

[3]Coral Reef Research Center of China, Guangxi University, Nanning 530004, China kefuyu@scsio.ac.cn

In the following we provide supplemental information about:

- Research sites on Hainan Island in southern China
- Temperature record over period in question

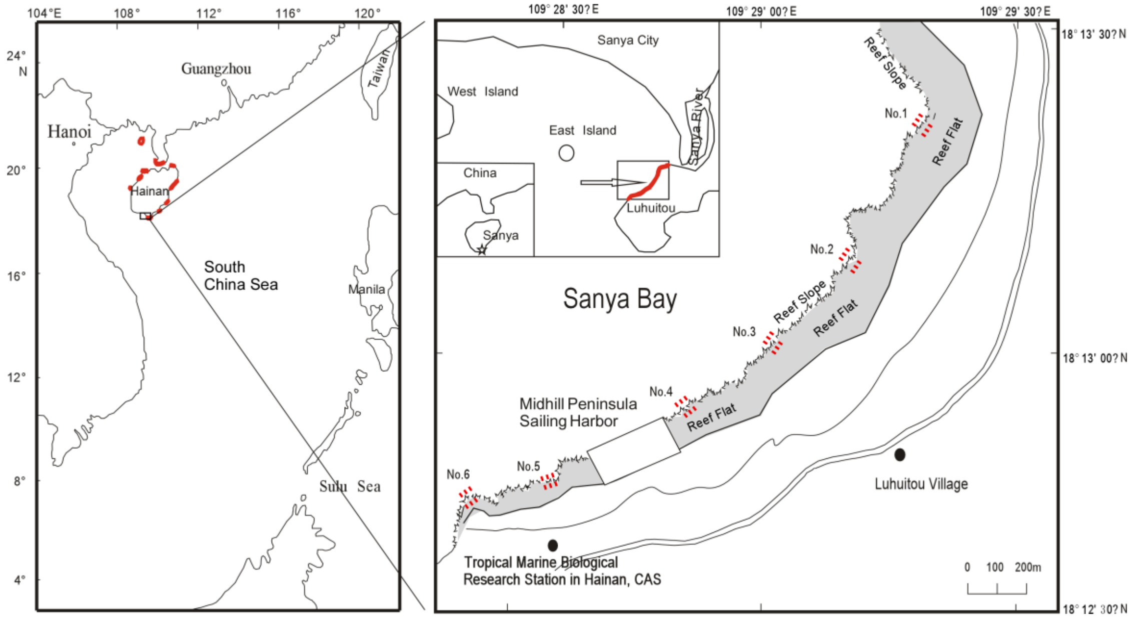

Figure S-1: Luhuitou Reef on Hainan Island, in the northern S-China Sea. Reef areas around Hainan shown in red. Transects study a habitat gradient at 2m on top of the reef flat (shown in gray) and at 4m, on the reef slope. Video transects (50-m long) used for the present study were taken at sites (dotted lines) parallel to the depth contours. Original map produced in Corel Draw (www.coreldraw.com) 14 by M. Zhao, continental and island outlines generated with Freeware R (www.r-project.org), using libraries "map" and "mapdata".

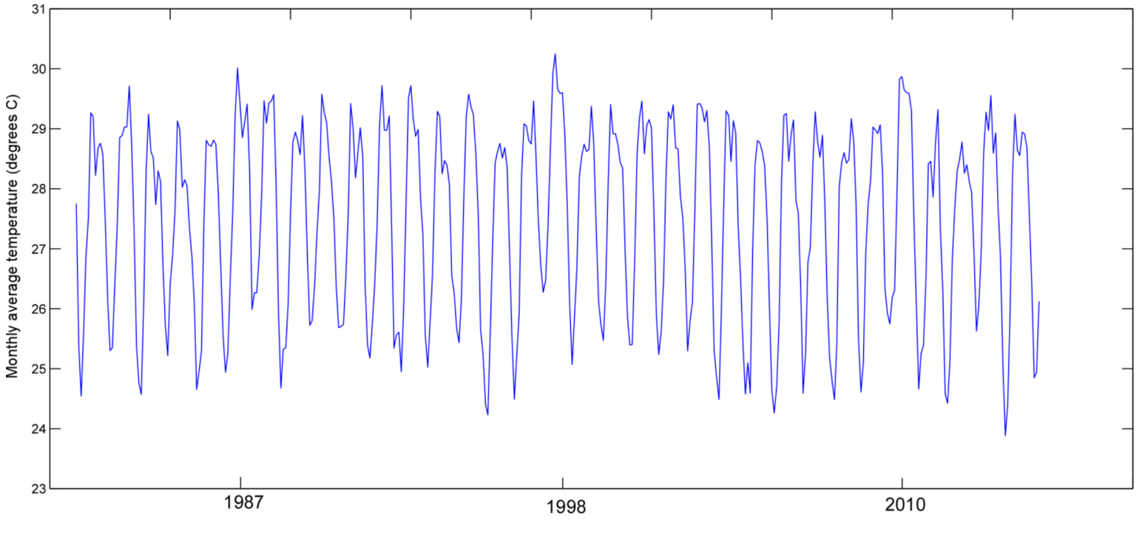

Figure S-2: Mean monthly temperatures in the temperature tile encompassing Luhuitou Reef on Hainan Island, in the northern S-China Sea. 1998 and 2010 have clearly higher monthly mean maxima, which is usually indicative of coral stress and, potentially, bleaching and mortality during this period. Temperatures are from OISST hindcast[1].

References:

1. Reynolds, R.W., Rayner, N.A., Smith, T.M., Stokes, D.C. & Wang, W. An improved in situ and satellite SST analysis for climate. *Journal of Climate* **15**, 1609-1625 (2002).
